# Machine learning‐based radiotherapy time prediction and treatment scheduling management

**DOI:** 10.1002/acm2.14076

**Published:** 2023-08-17

**Authors:** Lisiqi Xie, Dan Xu, Kangjian He, Xin Tian

**Affiliations:** ^1^ School of Information Science and Engineering Yunnan University Kunming China; ^2^ Department of Radiation Oncology The Second Affiliated Hospital of Kunming Medical University Kunming China

**Keywords:** machine learning, radiotherapy, time prediction, treatment scheduling

## Abstract

**Purpose:**

The utility efficiency of medical devices is important, especially for countries such as China, which have a large population and shortage of medical care resources. Radiotherapy devices are among the most valuable and specialized equipment categories and carry enormous treatment loads. In this study, a novel method is proposed to improve the efficiency of a radiotherapy device (linac). Although scheduling management with accurate prediction of the entire treatment time included in each appointment, arrange a reasonable time duration for appointments and save time between patient shifts effectively. Tasks belonging to the treatment and non‐treatment groups can be assigned more flexibly based on the availability of time.

**Material and methods:**

Data from 1665 patients, including patient positioning time (PT) and treatment time (TT), were collected in collaboration with the Radiotherapy Center of the Department of Oncology at the Second Affiliated Hospital of Kunming Medical University from November 2020 to August 2021. The features related to PT and TT were extracted and used to train the machine learning‐based model to predict PT and TT in independent patients. The prediction results were subsequently applied to a minute‐based scheduling tool.

**Conclusion:**

Artificial intelligence is a promising approach to solve abstract problems with a specialized knowledge background. The results of this study show encouraging prediction outcomes in relation to effective scheduling management and could improve the efficiency of the linac. This successful trial broadens the meaning of medical data and potential future research directions in radiotherapy.

## 1 INTRODUCTION

Cancer is among the most serious diseases worldwide, with a high level of morbidity and mortality.^1^, ^2^ In China, treatment load and medical care resources are unbalanced due to the large population, and local medical units are burdened with many cancer patients.^3^ This burden of providing healthcare to cancer patients demands a professional health workforce, advanced medications, and specialized devices for cancer management. According to public data, 50%−60% of cancer patients require radiation therapy as part of their treatment strategies.^4^ In other words, as one of the three (surgical treatment, chemotherapy, and radiotherapy) main approaches for cancer management, radiation therapy and the optimal use of radiotherapy resources play a significant role.

**FIGURE 1:**
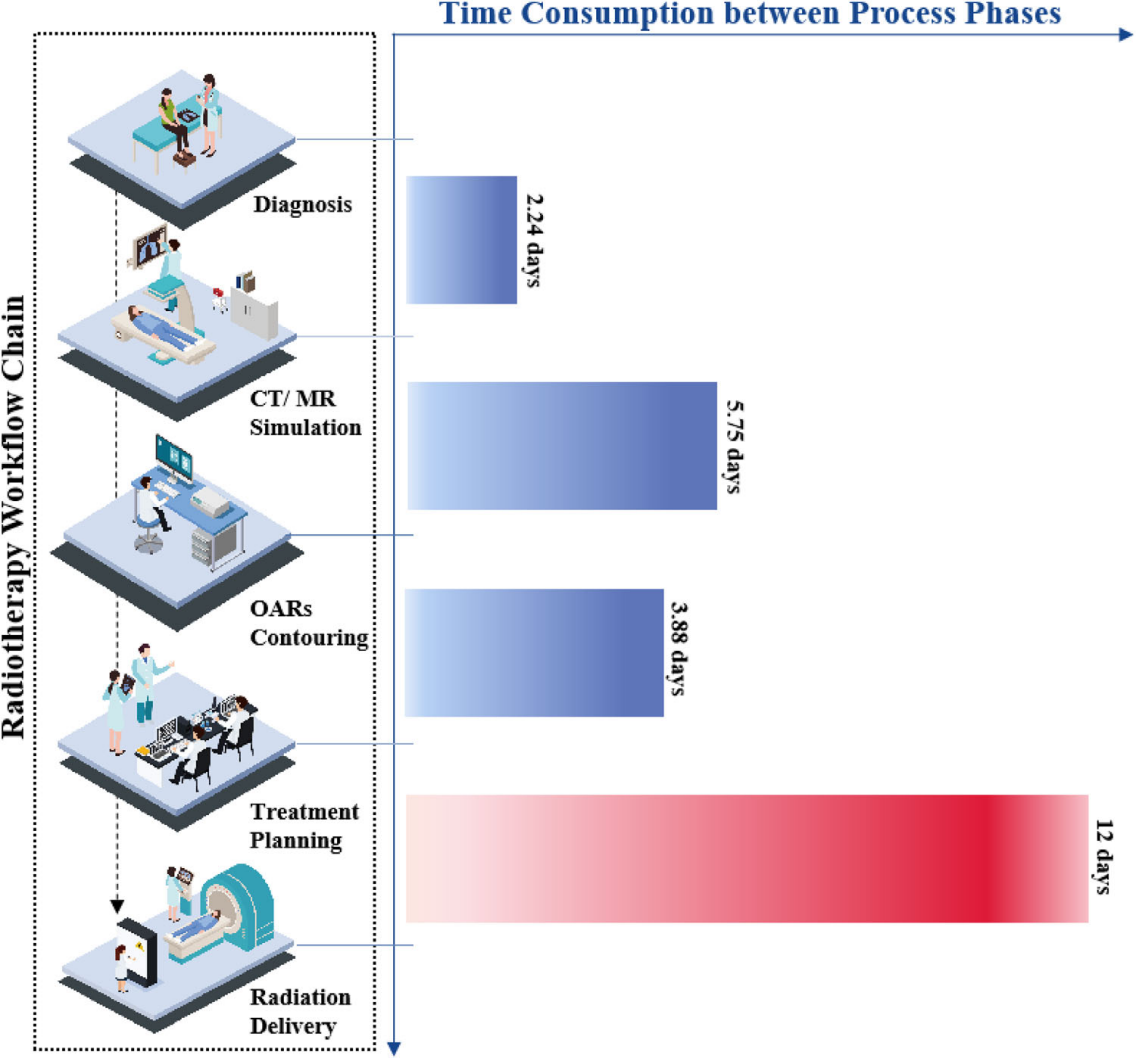
Time consumption between process phases in radiotherapy workflow chain.

**FIGURE 2:**
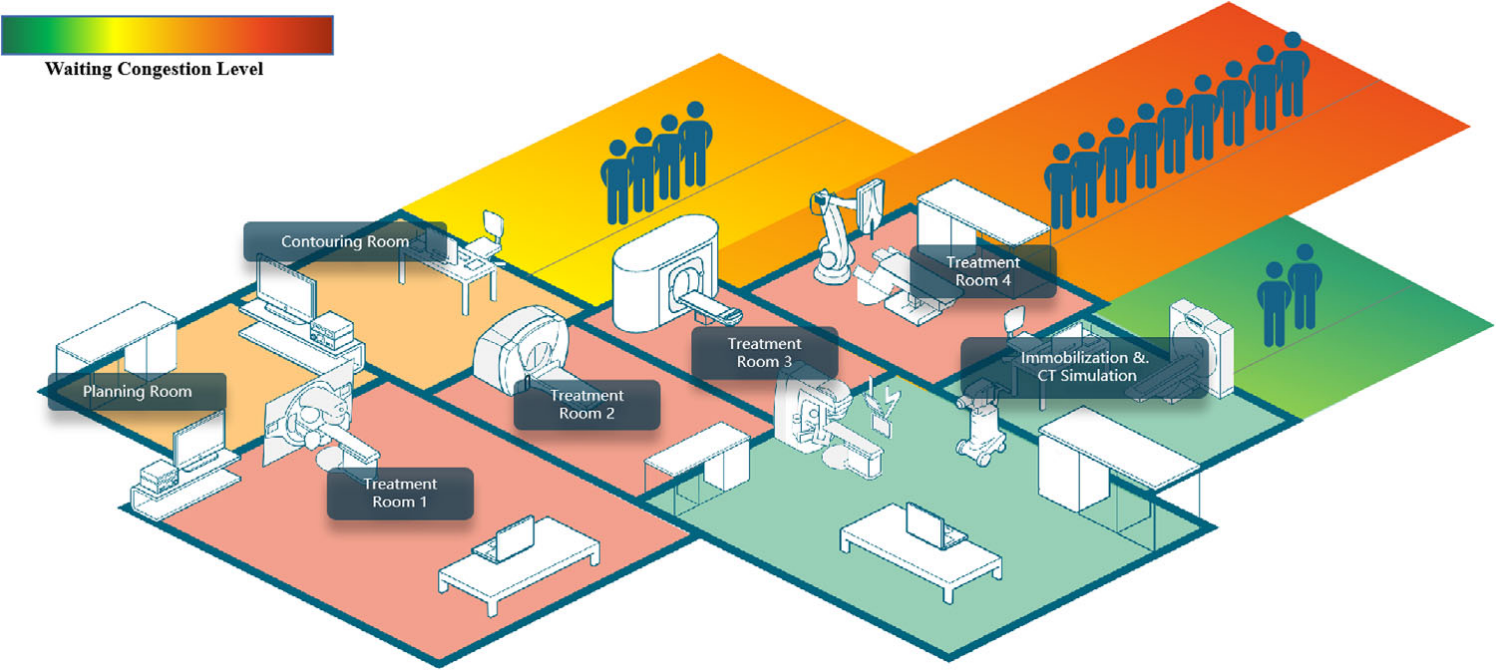
Efficiency diagram of different phases.

Many patients who receive radiotherapy must wait a long time to start the course, and delays in radiation therapy can affect the effectiveness of the overall cancer treatment regimen. For this reason, some countries have introduced limits on waiting time for patients scheduled to receive radiotherapy.^5^, ^6^ However little progress has been made due to the low efficiency of treatment, and one of the most crucial reasons is the lack of efficient scheduling management.^7^ As shown in Figure 1, based on a study reported by the National Cancer Center of China, the mean duration between different phases of the radiotherapy process in the current radiotherapy workflow is a few days,^8^ while the mean waiting time to the first irradiation lasts more than 1 week. This not only suggests a shortage of radiation devices in China, but also the importance of improving the utilization efficiency of these machines. Figure 2 illustrates the efficiency of each process/phase in the radiotherapy workflow. Radiotherapy can be performed in two ways: external irradiation using machines or internal irradiation using brachytherapy devices. Most departments of radiation oncology in China are equipped with linear accelerators. Therefore, the scheduling management of linacs (i.e., how the linac resources can be taken full advantage of) is the key problem to solve. In addition, improving the linac utilization rate has several benefits. First, the capability of linac in a single workday (i.e., the maximum number of patients per machine) can be increased. A significant amount of time is wasted during interpatient switching and this can be decreased by optimizing the treatment schedule. Furthermore, the workload (i.e., shifts or work hours) of radiotherapy specialists will be reduced if the machine is used more efficiently. More importantly, quality assurance for patients with acute cancer who undergo special radiotherapies [such as stereotactic body radiation therapy (SBRT), stereotactic radiation surgery (SRS), and adaptive radiotherapy (ART)] can be provided. However, the key point that affects appropriate treatment scheduling is an accurate estimation of the entire treatment time (TT) for each patient, including positioning. Factors that affect positioning and TT include cancer site, irradiation technique/mode, and immobilization method to position verification approach, the delivery dose per fraction to the dose rate that the performing linac can afford, likewise, if this is the first treatment fraction to whether the Image guidance (IGRT) is required.

For this reason, multiple elements should be considered when assigning a specialized time slot to a patient during scheduling. However, current common strategies for radiotherapy scheduling include “as soon as possible,” linear programming, online stochastic algorithms, etc.,^9^, ^10^, ^11^ and they do not always rely on precise time estimation‐based division. To our knowledge, no generally accepted guideline matches the predicted treatment duration for each patient to personalized treatment plans. In this study, a novel machine learning (ML)‐based scheduling method for radiotherapy is proposed. Addressing the scheduling problem by accurate prediction of individual treatment times, which are subsequently applied to time slot assignment was the mainstay of this approach. It comprises three steps. In the first step, by splitting the entire treatment into subphases, factors related to the duration of patient positioning and treatment execution were extracted, analyzed, and classified into a standardized group. Next, we built a real‐data‐based prediction model for total treatment duration using the ML method. The treatment duration in this study was divided into two parts: positioning time (PT) and TT. Using the ML method, both PT and TT are precisely predicted in minutes orders of magnitude, so that these data can be further used in the third step, which calculates all available time in the schedule in minutes orders of magnitude. With precise utilization of the occupied and available time of the linac, scheduling management shows a more efficient and flexible performance. Tasks such as treatment, quality, and maintenance that affect radiotherapy durations, can be arranged reasonably.

## 2 MATERIALS AND METHODS

In this study, we collaborated with the radiotherapy center of the Department of Oncology at the Second Affiliated Hospital of Kunming Medical University. We collected data on individual treatment duration for each patient's workday, while the characters of positioning and parameters of the radiotherapy plan coordinate were recorded from November 2020 to August 2021, with 1665 data points in total. This radiotherapy center has one linac, Infinity (Elekta Solutions AB, Stockholm, Sweden), which serves approximately seven million people in Kunming as one of the nine linacs in this area. The average treatment task of the radiotherapy center is approximately 60 patients daily, suggesting that approximately 1200 patients cover all subtypes of cancer annually.

Radiation therapy is a continuous course since fractionated prescription doses are administered on sequential days during the treatment period. As a result, the time of the first treatment fraction becomes the direct factor affecting the timeliness of cancer management. Several studies have revealed negative effects induced by a delay in starting radiotherapy.^12^ However, the cases in our study received a waiting time between 1 and 3 weeks from the radiotherapy plan confirmed by the oncologist to the irradiation of the first treatment fraction. The reasons for this are summarized as follows: (a) Overall fractions ranged from 25 to 33 fractions for each patient according to the prescription, unless the lower fractional radiotherapy is chosen, which is only in the case where SBRT/SRS is needed. Meanwhile, the duration of a single treatment is distributed from 5 to 25 min, depending on different immobilization and positioning approaches, as well as the parameters corresponding to dosimetry delivery and irradiation technologies. In other words, the treatment schedule is generally filled with a relatively fixed number of appointment slots sustained over a period of time. The newly confirmed radiotherapy plan can only be arranged in the treatment schedule either until the treatment phase belonging to one of the previously listed patients is completed or the work hours of the therapist are extended to accommodate these additional cases. (b) Two common strategies are applied in the definition of the time slot for radiotherapy scheduling: block and non‐block methods. In our case, the Department of Oncology at the Second Affiliated Hospital of Kunming Medical University adopted the block method, in which the therapists divided daily work hours into several sections and divided each section into four slots with the same length of time. A slot is occupied when an appointment is accepted; similarly, the same slots in the coming successive workdays are locked for this patient. It leads to a notable waste of time since the treatment duration greatly differs as patients change. The linac must remain idle when adjacent patients take less treatment time than in slot settings. In contrast, patients in later queues have to manifold wait as plans over time are placed intensively along the slot queue, let alone leave available time for new cases. (c) For specialized radiotherapy techniques, such as SBRT/SRS, and personalized management, such as ART, because the fractional dose and irradiation accuracy are increased, strict quality assurance (QA) is forced before treatment, which also occupies machine resources. In addition to patient QA, several activities, such as QA and device maintenance related to linac operation, must be considered in the time assignment of the linac. Table 1 shows the relevant activity items, definitions, and time consumption for execution regarding the linac.

**TABLE 1:** Items, definitions, and time consumption within the available time per linac unit.

Usage aim	Category	Object	Time consumption	Definition
Treatment	Treatment	Non‐first fraction	15−22 min/patient	Duration of treatment for patients in the non‐first treatment fraction, workdays.
		First fraction	25−30 min/patient	Duration of treatment for patients in the first treatment fraction, workdays.
Non‐Treatment	Machine QA	Daily QA	10−15 min/workday	The accuracy of radiation and energy is measured daily before treatment, workdays.
		Weekly/monthly/annually QA	2−4 h/execution	Machine QA is performed according to different frequent requirements, usually on weekends.
	Patient QA	Dosimetry verification	5−8 min/patient	Dosimetry verification of plans must be completed before the first treatment fraction, on weekends or workdays.
	Maintenance	Breakdown repairs	N/A.	Only needed in case of equipment breakdown.
		Routine maintenance	1−1.5 h/time	Routine maintenance, on weekends.
		Periodic maintenance	2 h/time	Regular device care, on weekends.

In this study, an auto‐machine learning (auto‐ML) method is introduced, and the model for time prognosis is trained based on credible data. Therefore, the slots in the schedule are redefined as the predicted length of time instead of a fixed setting, in which way the utilization rate of linac can be greatly improved; thus, the saved time after optimization can be operated flexibly for additional cases, QA tasks, regular maintenance, and clinical scientific research purposes.

### 2.1 Features extraction

In the preparation stage, data on the overall treatment duration for an individual patient were investigated following the diagnosis and irradiation plan to determine characteristic factors that potentially affect the length of positioning and treatment duration, respectively. Specifically, the entire treatment of one fraction is divided into PT and TT. For PT, actions are further separated into patient immobilization and positioning verification, in which the immobilization method, immobilization site, and implementation of specialized ancillaries are elements in which the positioning duration differs among patients. Similarly, for TT, the duration depends on the complexity of the treatment plan, which can be based on analysis quantized as factors including the treatment site, irradiation dose per fraction, irradiation technology, and number of control points. Table 2 shows the feature factors extracted from the patient positioning and treatment phases.

**TABLE 2:** Feature factors extracted from patient positioning and treatment phase.

Time Category	Feature Category	Feature Object		Definition	Value
Positioning time	Patient Immobilization	Immobilization site		Site to immobilize	Head/head&neck/thorax/breast/ pelvic
		Immobilization method		Which immobilization board is applied	Head/head&neck/thorax/breast/ pelvic/SBRT
			Six‐dimension treatment table	If a six‐dimension treatment table is requested	Yes/no
		Specialized ancillaries	Vacuum cushion	If a vacuum cushion is requested	Yes/no
			Metallic implant	If a metallic implant is requested	Yes/no
	Immobilization Verification	IGRT		CBCT/EPID	IGRT/Non‐IGRT
Treatment time	The complexity of the treatment plan	Treatment site		Site to irradiate	Head/head&neck/thorax/breast/ pelvic
		Irradiation technology		Which Radiation technology board is applied	2D/3D‐CRT/IMRT/VMAT/SBRT
		Prescription dose		Irradiation dose per fraction	2−12 Gy
		Number of control points		Number of segments	0−1024

To guarantee that all feature data for modeling are reliable, all related terms are normalized through extraction from the Radiotherapy Planning System Monaco (Elekta Solutions AB, Stockholm, Sweden), Oncology Information System Mosaiq (Elekta Solutions AB, Stockholm, Sweden), and Radiotherapy Workflow Management System (RWMS) Via (Leg Limit., Kunming, China), recorded, and further used in ML model training. Table 3 shows the information systems involved in the sources of feature data acquisition.

**TABLE 3:** Source of feature data acquisition.

Time category	Feature object	Data source (information system)
Positioning time	Immobilization site	Via Info sys.
	Immobilization method	Via Info sys.
	Six‐dimension treatment table	Via Info sys.
	Vacuum cushion	Via Info sys.
	Metallic implant	Via Info sys.
	IGRT	Mosaiq
Treatment time	Treatment site	Mosaiq
	Irradiation technology	Monaco
	Prescription dose	Mosaiq
	Number of control points	Monaco

The time duration of PT and TT were collected using RWMS, with the start and stop times of each step automatically recorded. As a treatment appointment began, therapists were enabled to access the patient's treatment plan to check personalized positioning requirements and performed appropriate positioning operation accordingly. The procedure was turned to “Irradiation” step when “Positioning” is complete. The “irradiation” process lasted differently depending on individual radiotherapy plan executed. Since the “start” and “complete” time points of “Positioning” and “Irradiation” step were automatically recorded in RWMS, the duration for PT and TT were then automatically calculated and qualitatively analyzed. All PT and TT data involved in our study were calculated automatically from RWMS, in which the timing system is a standard calendar system with year, month, day, hour, minute, and second. There were no decimal points for PT and TT times since all times data are calculated in “seconds” which in coordinate with the minimum recording unit in timing system.

### 2.2 Auto‐ML model training

**FIGURE 3:**
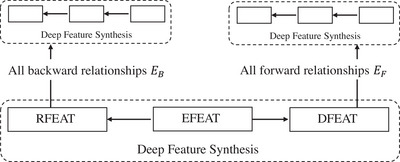
The workflow chart of DFS.

In this study, auto‐ML was adopted to predict PT and TT in radiotherapy.^13^, ^14^, ^15^ Auto‐ML comprises four steps: feature extraction, model building, model optimization, and evaluation. In the first step, we used the deep feature synthesis (DFS)‐based method to process the source data. A flowchart of the DFS is shown in Figure 3.

ALGORITHM 1 **Generating features for the target entity**

**Table:** 

function $Make\_feature{\left(E^{i},E^{1:M},E_{V}\right)}_{)}$
$E_{V}=E_{V}\cup E^{i}$
$E_{B}=Backward\left(E^{i},E^{1,\text{…},M}\right)$
$E_{F}=Forward\left(E^{i},E^{1,\text{…},M}\right)$
for $E^{j}\in E_{B}$ do
$Make\_feature(E^{j},E^{1,\text{…},M},E_{V})$
$F^{j}=F^{j}\cup rfeat\left(E^{i},E^{j}\right)$
for $E^{j}\in E_{F}$ do
if $E^{j}\in E_{V}$ then
Continue
$Make\_feature(E^{j},E^{1,\text{…},M},E_{V})$
$F^{i}=F^{i}\cup dfeat\left(E^{i},E^{j}\right)$
$F^{i}=F^{i}\cup efeat\left(E^{i}\right)$

In Figure 3, $E_{B}$ denotes the backward relationship tables and $E_{F}$ is the forward relationship tables in a given dataset $E^{1,\text{…},K}$, where each entity table has 1,…,J features. The details of generating features for the target entity are provided in Algorithm 1.

In Algorithm1, dfeat denotes the direct features applied over the forward relationships. efeat denotes the entity features, which derive features by computing a value for each entry $x_{i,j}$ by

(1) $$x_{i,j^{\prime }}=efeat\left(x_{:,j},i\right)$$

rfeat denotes the relational features, which are applied over the backward relationships from $E^{l}$ to a new feature $E^{k}$ by

(2) $$x_{i,j^{\prime }}^{k}=rfeat\left(x_{:j|e^{k}=i}^{l}\right)$$

In model building, we used the following optimization function as the model selection basis:

(3) $$A^{*}\in \arg\min_{A\in A}\frac{1}{k}\sum_{i=1}^{K}L(A,D_{train}^{\left(i\right)},D_{valid}^{\left(i\right)})$$

where $L(A,D_{train}^{\left(i\right)},D_{valid}^{\left(i\right)})$ denotes the loss of model A on the training set $D_{train}^{\left(i\right)}$ and validation set $D_{valid}^{\left(i\right)}$.

After selecting the ML model, we used the following function to optimize the hyperparameters of the selected model:

(4) $$\lambda^{*}\in \arg\min_{\lambda \in \Lambda }\frac{1}{k}\sum_{i=1}^{K}L(A_{\lambda },D_{train}^{\left(i\right)},D_{valid}^{\left(i\right)})$$

where $\lambda_{1},\text{…},\lambda_{n}$ is the hyperparameter that must be set in A, and A denotes the ML model and $A=\{A^{\left(1\right)},\text{…},A^{\left(k\right)}\}$.

To demonstrate the time prediction accuracy of the proposed method, we use the accuracy in the range T (in seconds) of true treatment time. The prediction accuracy in the range T (in seconds) is calculated by

(5) $$acc=\frac{1}{n}\sum_{p=1}^{n}x_{p},\left\{\begin{array}{c} if\left|x_{p}-x_{T}\right|\leq T,x_{p}=1\\ else,\;x_{p}=0 \end{array}\right.$$

where $x_{p}$ is the predicted time of case P and $x_{T}$ is the real time of case P.

## 3 RESULTS

### 3.1 Predicting PT and TT

In this section, all positioning and treatment data are from real radiotherapy data provided by our cooperating institution, the radiotherapy center of the Department of Oncology at the Second Affiliated Hospital of Kunming Medical University (SAHKMU). We collected 1665 radiotherapy samples in total and used 1165 of them as training data and the remaining 500 samples as testing data.

To prove the prediction performance of the proposed model, a multiple linear regression (MLR) time prediction model was used in most hospitals as a baseline for experimental comparison.^16^, ^17^, ^18^ Most hospitals use the following models to predict PT:

(6) $$Y_{p}=C_{1}+\alpha_{1}X_{1}+\alpha_{2}X_{2}+\alpha_{3}X_{3}$$

where α denotes the weights of different parameters, X_1_ denotes the coding of the treatment site, and X_2_ denotes whether the current treatment is the first time. X_3_ is a technique used in radiotherapy.

The TT can be predicted by:

(7) $$Y_{t}=C_{2}+\beta_{1}X_{1}+\beta_{2}X_{2}+\beta_{3}X_{3}+\beta_{4}X_{4}$$

where β denotes the weights of different parameters and X_4_ denotes the modulation of the radiotherapy machine. By calculating the data, the weights in Eqs. 5 and 6 are as follows: C_1_ = 3.551, α_1_ = −0.0154, α_2_ = 0.18287, and α_3_ = 0.3846. C_2_ = 3.5518, β_1_ = −0.0569, β_2_ = 0.3962, β_3_ = 0.0014, β_5_ = 0.496.

**FIGURE 4:**
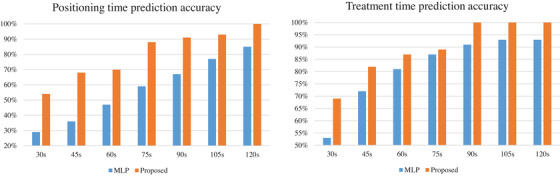
Bar chart of time prediction accuracy comparison.

Table 4 shows a comparison of the MLR and the proposed method with respect to positioning accuracy and treatment accuracy. It can be seen that the proposed method outperforms the existing MLP methods in both positioning and treatment prediction accuracy. Within 60 s, the prediction accuracy of the proposed positioning and TTs reached 81% and 87%, respectively. Within 90 s, the prediction accuracy of the treatment time was 100%. Figure 4 shows the prediction time comparison between the MLP and the proposed method. The times from 30 to 105 s in first row mean the error range between the predicted values and the real values of test data.

**TABLE 4:** Analysis of time prediction accuracy.

Time range (/s)		30 s	45 s	60 s	75 s	90 s	105 s
Positioning	MLR	29%	36%	47%	59%	67%	77%
Accuracy	Proposed	53%	72%	81%	87%	91%	93%
Treatment	MLR	54%	68%	70%	88%	91%	93%
Accuracy	Proposed	69%	82%	87%	89%	100%	100%

As seen in Figure 4, our prediction accuracy has a prominent advantage over the traditional MLP‐based method in the small‐time error range, such as 30, 45, and 60 s. Figure 4 also shows the potential of the proposed method in real radiation therapy, which can effectively save time and improve scheduling efficiency.

### 3.2 Scheduling management tool based on the prediction result of auto‐ML

**FIGURE 5:**
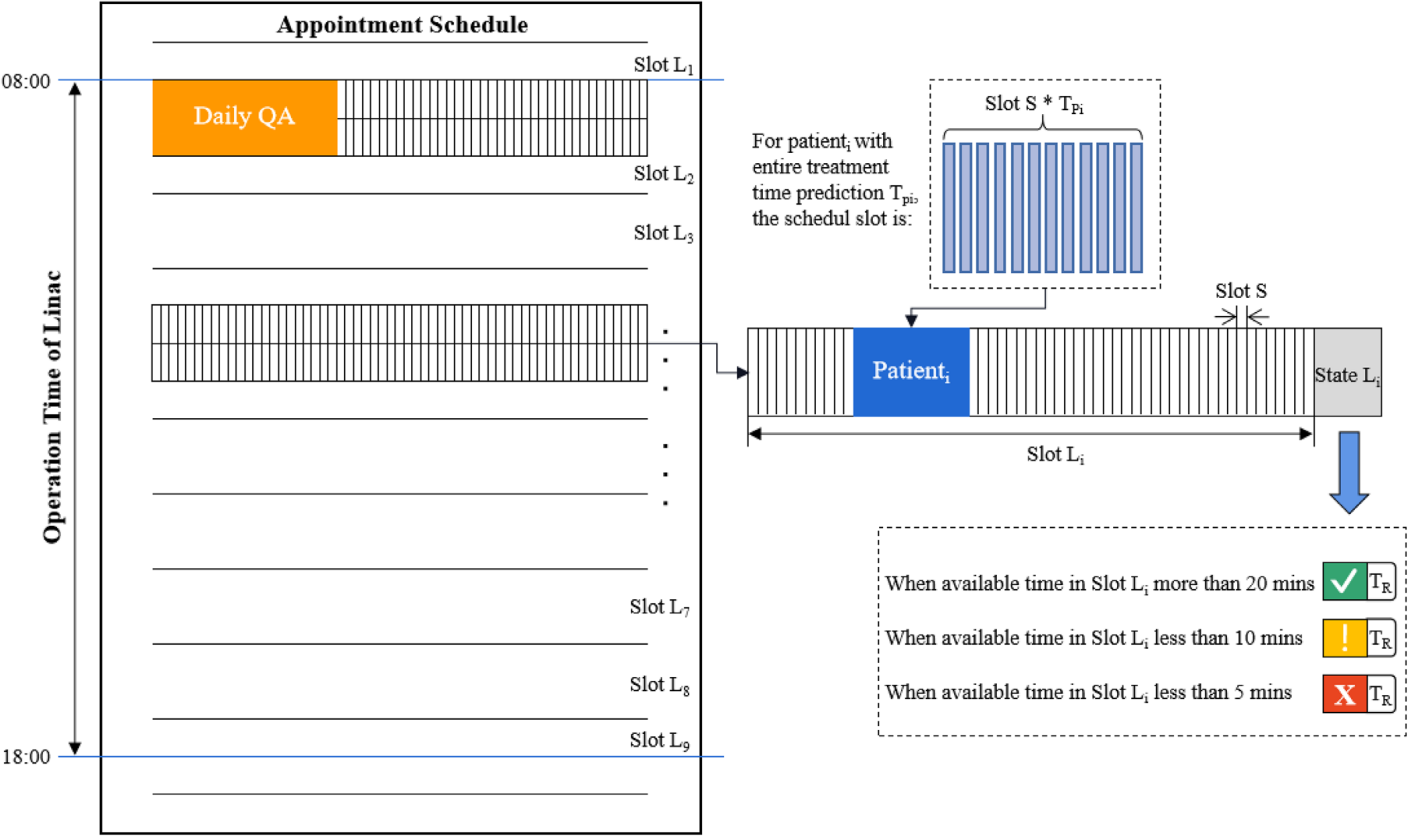
Diagram of scheduling management tool with prediction result based on Auto‐ML.

Based on the prediction results using the auto‐ML algorithm introduced in Section 2.2, an improved scheduling management tool was designed to organize treatment appointments accurately for minutes. Figure 5 illustrates the principle of the scheduling procedure. As shown in Figure 5, the treatment appointment schedule is divided into several large time slots, “Slot L,” during the operation time of the linac. The length of “Slot L” depends on the shift system of radiotherapy technicians/therapists or the reservation time of patient groups. For each “Slot L_i_,” equal amounts of unit slot “Slot S” with an identical length of 1 min were further divided. Therefore, the predicted entire treatment duration for each patient occupies the corresponding length of “Slot S” when scheduling. A simple example is provided at the Radiotherapy Center of the Department of Oncology at SAHKMU. The operation time of the linac at SAHKMU is from 08:00 a.m. to 18:00 p.m., 10 h in total, during which ten 1‐h‐sized “Slot Ls” are divided. Daily QA, including dosimetry and mechanical accuracy checks for linac before the treatment of the first scheduled patient, is mandatory at SAHKMU, which takes approximately 20 min. Except that, altogether, 520 min are available to arrange patients’ appointments, with 60 “Slot Ss” included in each “Slot L_i_.” As scheduled patients accumulate, the rest time, namely, the available “Slot S,” is important since it indicates which kind of tasks are suitable to assign without affecting the next “Slot L.” Therefore, at the end of each “Slot L_i_,” an indicator “State L_i_” is placed to show the capacity of rest time in “L_i_.” Three indication colors, “Green,” “Orange,” and “Red,” are adopted, which means “available time in Slot Li more than 20 min,” “available time in Slot Li less than 10 min,” and “available time in Slot Li less than 5 min,” respectively. When an emergency case is needed, it helps adjust the treatment order with a clear indication of the rest time and length of tasks. In addition, the combination of tasks with different magnitudes prevents frequent errors or breakdowns by avoiding intensive long‐time sustaining irradiation.

The scheduling tool proposed in this paper was applied for testing in the collaborated radiotherapy center: the Department of Oncology at the Second Affiliated Hospital of Kunming Medical University. The testing period last four months, during which circa one hour in average in each workday was saved from 10 work hours in total when arranging same quantity number of treatment appointments. Namely 10% optimization of scheduling efficiency was achieved as the proposed scheduling tool was applied. Meanwhile, with the saved work hours, six to eight new patients can be treated on single linear accelerator additionally.

## 4 DISCUSSION

Scheduling management, a newly recognized problem in precise radiotherapy, has been of considerable interest in recent years. However, in several studies, the time spent on pretreatment preparation is the only problem that has received the most attention,^8^, ^19^ while the efficiency of scheduling management, that is, the utility rate of the radiation device, is ignored. In this study, realistic clinical work was performed more appropriately.

The entire treatment duration of each patient was divided into two parts for analysis: PT and TT. Features characterizing the duration of positioning and treatment are extracted correspondingly from a standardized RT information system, according to which a neural network algorithm is subsequently built to predict PT and TT separately. As the estimated positioning and TTs are calculated independently using the auto‐ML model, the overall treatment duration prediction for an individual patient is equal to the sum of these two results and is further used as a novel scheduling arrangement tool that manages the available working hours of the linac accurately to minutes.

Our proposed time prediction method and scheduling management tool won the second prize at the 5^th^ National Smart Health Innovation Final Competition (SHIC 2022)^20^ and the 1st Medical Information Innovation Conference of China. Our study strongly indicates that a linac can be fully used for treatment intention by increasing 20%−30% more patients in regular work hours, depending on the duration of new cases, which indicates that the prediction results can precisely reflect the realistic length of the entire TT so that the arrangement of schedule slots can be guaranteed. The robustness of the scheduling management method is significantly higher than that of scheduling management previously reported, which is assisted by the prediction of future workload,^21^ because the capability to control dynamic cases and events are enhanced by implementing an online scheduling adjustment mechanism. This appears to be similar to those reported by Legrain et al. and Chang et al., who used an online stochastic algorithm to deal with dynamic treatment tasks,^10^, ^22^ but the improvement of our study is notable because the neural network is introduced to generate a highly precise estimation of the entire TT for each patient. Based on the dynamic scheduling management mechanism, the combination with auto‐ML strengthens the scheduling accuracy.

In addition, from the perspective of the patients, by improving the linac utility rate, a shorter waiting period is needed until their first fraction of RT treatment starts. Therefore, tumor progression can be maintained at a stable level, which increases the stratification level of patients. Meanwhile, the time saved by the improved efficiency of linac can benefit both clinical and non‐treatment aspects. According to the data analysis in our research, generally, technologies such as complicated positioning methods and the application of IGRT that are enforced in modern precise radiation strategies obviously cost longer. The high efficiency of the linac provides more time to implement these technologies. In addition, non‐treatment tasks, such as machine QA, device maintenance, and clinical scientific trials that occupy machine resources, can be arranged more flexibly and reasonably. Moreover, in this study, the visualization of duration length is proposed as a novel solution to manage appointments. With a combination of appointments with time lengths of different orders of magnitude, intensive workloads for linacs are inevitable.

## 5 CONCLUSION

The last ring of the radiotherapy chain is the dose irradiation, namely, the execution of radiotherapy plan, which generated after previous operations in radiotherapy chain: diagnosis, CT image acquisition, ROIs (Regions of Interests) contouring and treatment planning design. Therefore, as the last ring of the radiotherapy chain, radiation is of great significance, highlighting both the timeliness and efficiency of appointment management. This is particularly true when there is an imbalance between medical resources and treatment loads. In this study, a scheduling management method based on the ML algorithm is the first time proposed, in which the ML model is trained to predict highly precise results of individual treatment time per fraction, and the predicted time is further used in the arrangement of appointments. According to the results, artificial intelligence has been successfully used to solve prediction problems affected by various abstract features and to inversely assist clinical practice. By implementing the encouraging predicted time for scheduling management, the utility rate of the linac is improved, which results in a more reasonable assignment of work hours of linac, both for treatment and non‐treatment intentions. The guarantee of the necessary QA and maintenance of the linac ensures that the device can perform RT plans with satisfactory accuracy for dose delivery and mechanics. Similarly, it helps to maintain the linac in a sustainable operating state. Another advantage revealed in this study that benefits the life cycle care of treatment machines is the combination of treatment plans with time lengths of different orders of magnitude by appointment arrangement. This leads to decreased machine errors and breakdowns caused by intensive irradiation for a long time. Finally, this study strengthened the value of medical data generated during the treatment process. Although many similar data types are produced discretely, separately, and independently, the development and implementation of various RT information systems provides mature platforms to collect standardized data. Once these ignored medical data are fully utilized through information technology, they have great potential for future and further scientific research.

## AUTHOR CONTRIBUTIONS

All authors contributed significantly to the performed work and approved the manuscript for publication.

## CONFLICT OF INTEREST STATEMENT

The authors declare that there are no conflicts of interest regarding the publication of this article.
